# A Treatise on the Anatomy of Regions

**Published:** 1832-04-01

**Authors:** 


A TREATISE ON SURGICAL ANATOMY; OR THE ANATOMY OF REGIONS, CONSIDERED IN ITS RELATIONS WITH SURGERY. ILLUSTRATED BY PLATES, REPRESENTING THE DIFFERENT REGIONS OF THE BODY. By *Alf. A. L. M. Velpeau*, M.D.P. Agrégé Stagiaire to the Faculty of Medicine of Paris, &c. In Two Volumes. Translated from the French, with additional Notes, by *John W. Sterling*, M.D. M.R.C.S.L. &c. &c. &c. Pp. 456, 523. Octavo, New York, 1830.

WE have just received this translation, and have not had an opportunity of seeing the original work, published in the French language by M. Velpeau. Its size and nature will prevent our attempting any thing like an analysis or review, but we think it due to the indefatigable author, and indeed to the public, to put them in possession of the objects and general character of the volumes. Nothing of the kind has hitherto been tried on a comprehensive plan, though detached descriptions of the surgical anatomy of particular systems and parts, have been published at intervals and received with much favour by professional students. As instances of this, we may refer to the work of Mr. Harrison, of Dublin, upon the arteries, of Allan Burns upon the head and neck, of Dr. Colles on the thorax, abdomen, and pelvis. It was the same abroad, and though many French surgeons had projected works of the present description, and some had even delivered lectures wearing all its features, none have yet given their systems to the public, with the exception of our present author. The differences between general, special or descriptive, and surgical anatomy are well pointed out by M. Velpeau in his preface.

"To examine the organic systems and whatever they possess in common in every part of the body, is the object of general anatomy; to study the apparatuses in succession; to describe the figure, volume, position, density and composition of each organ is the province of descriptive or special anatomy; to take a certain portion of the economy, describe all the elements which are comprised within it, and point out the peculiarities which each of them present; the direction and exact relations of the most important objects; the varieties of thickness and position produced by diseases or aberrations of development; to proceed from the skin towards the bones, or from the bones towards the surface, and thus observe successively, and layer by layer, in their relative and natural position, the different parts, without entering into minute details; this is what constitutes the anatomy of regions or of relations, or topographical anatomy.

The first, more particularly concerned with the fibrillary arrangement, and the analysis of the intimate structure of the tissues, is the basis of all sound physiology; without it, medicine would never have emerged from that confusion of principles which so long prevailed in the schools: it truly deserves the title of medical anatomy.

The second, displaying the organs in the manner which nature presents them, describing their most prominent characters, without investigating their molecular disposition, or those unknown vital properties from which they derive life and motion, appertains, more directly to surgery, which owes to it its rapid progress and the certainty with which it is honoured; without it, the surgeon would be but a dangerous man.

The third is as yet altogether new, and can only be considered as a complement of the two others. It differs from common descriptive anatomy, both by the end which it proposes and the means it employs. This takes up one apparatus of organs and follows it to every part to which it is distributed, previous to taking up the consideration of the others; that, on the contrary, passes in review all the elements of a circumscribed point, without investigating either their origin or termination. The one tends to make known the special functions of the economy; the other to expose the different characters of this or that part of the body; to give the mechanical reason for the diverse phenomena which we remark in it; to explain the difference in the dangers and forms of diseases, by the difference in the relative and visible disposition of the systems which compose this or that region; it dwells upon some organs, passes lightly over others, always seeks to place itself in relation with operations; in a word, it is the anatomy which is most intimately connected with external pathology, and which, for this reason, is called *Surgical*." vi.

The objects of the author cannot be more briefly stated than they are by himself in the latter part of the Preface.

"I have had no intention of making a treatise on anatomy to supply the place of those which we already possess, nor a book of surgery nor of operations, but to collect in each point of the body of man the knowledge which naturally flows from the parts which we there meet with: to propose an anatomy, by the aid of which the surgeon may always foresee, previous to practising any operation whatsoever, all the accidents which may immediately follow it, and all the precautions which it requires relatively to the parts which should be preserved or avoided; by the aid of which, one point of the body being given, it will be possible to tell, within a few lines, what are the fibrillæ, arteries, veins, nerves, muscles, etc., which must lie in the way of the instrument; and it was with the view of accomplishing this end still more effectually, that I was disposed to describe from the central point to the periphery, upon sections made at different heights of the trunk and extremities, all the objects which present themselves to the eye. I was also inclined to traverse the body in a great number of directions and at different points, with metallic rods, in order to indicate, by leaving them in place, the nature and situation of the organs thus transfixed; but I was apprehensive of making the work too voluminous, and therefore have reserved this project until a future opportunity." xiv.

If these promises are kept, and the student presented with what they give him reason to expect, we need hardly say that the work is calculated to be highly beneficial to the science of surgery. We cannot pretend, of course, to read with care a thousand pages in order to pronounce a critical opinion on the merits of an anatomical treatise; this would be imposing too hard a burthen even on a reviewer. But in France, where there has been more time, and probably sufficient inclination, to criticise, certain objections have been urged against the execution, which appear to us to be not only candidly but also satisfactorily answered by M. Velpeau, in the preface to his second volume, published it would seem, at a period subsequent to the first. The objections are stated and met seriatim.

"1st. Some persons, whilst they praise the general distribution of the work, have objected that, in order to understand it, a knowledge of anatomy is necessary, and consequently, that it is not suited to students commencing the study of medicine. To this I reply that my intention was not to take the place of other treatises on anatomy, nor to make a book for those who have not yet acquired some knowledge of the organization of man; but that the surgeon might, by studying successively the different sections which I have established, obtain as exact and perhaps more positive information than by any other method.

2nd. Others, on the contrary, consider that I have erred in describing all the parts of the body, because a great number of them present but little surgical interest. This censure does not appear to me to be just. Indeed it was either necessary to make a complete treatise, or none at all. Now, in the first case, all the regions must necessarily be passed in review, the one after the other; and besides, is there one of them upon which we may not be called to perform some operations?

3d. Some have found the work too voluminous; and one asserts that I have dwelt too long upon surgical considerations, or pathological observations, whilst another says that I have insisted too much upon anatomical detail. Such censures annul one another, and I must confess that they have surprised me, in every way. In the first place, I dare affirm that it is impossible to treat the same subject in a single volume, without doing it superficially; unless we imitate those who make it their business to transform every good book into simple manuals; as if every one was endowed like Hippocrates, Boerhaave, Stoll, with the capability of reducing any part of medicine whatsoever to a few aphoristic sentences! Has not Burns published an entire volume upon the head and neck only? Those who read my production with some attention will be convinced, if I do not deceive myself, that I have not dwelt upon anatomical descriptions, except when it became necessary in order to explain certain methods of operation, some morbid phenomena, or when the parts did not seem to have been presented under their proper aspect. On the other hand, I beg leave to remark that the principal object of this work is to illustrate the practice of operations; consequently, I could not dispense with discussing the relative value of operatory processes founded upon the anatomical disposition of the parts.

4th. I am also censured for not having described more fully the external configuration, or picturesque anatomy. I admit that it would have been possible to have entered more into detail on this subject; but writing for surgeons rather than for painters, I was apprehensive that I had already gone too far in this respect. It would have been very easy, however, to have filled up this void, as it is the most simple part of the subject.

5th. There are those who have supposed that the regions might have been better designated from the presence of some important organ, than by arbitrary lines. Thus, they would have preferred *sterno-mastoidean, carotidean, laryngeal, tracheal, lingual, tonsillar,* &c. regions. This was the method which I attempted first: it would have exacted much less labour; but I soon perceived that it would render the objects extremely vague, and that all precision would have been impossible. Besides, it would only have been tracing the steps of *Winslow* and of *Malacarne*, and would have produced a work quite as useless as that of the latter author.

Furthermore, this plan seems to me to deviate too much from the rigid accuracy which ought to characterize surgical anatomy, for any one to adopt it, and I may therefore dispense with exposing the defects of it.

Finally, all these objections relate to the method only, and I observe with satisfaction that none have as yet been raised against the correctness of the descriptions or the judiciousness of the practical applications, which must essentially constitute the fundamental part of the work." vii.

The first volume contains the surgical anatomy of the head—neck—thoracic extremities—chest. As a specimen of the mode of subdivision, we may take the first chapter, that on the head. This is split into two articles; —1. On the Cranium—2. On the Face. Each of these parts is again subdivided into regions; the head into the frontal region—temporo-parietal region—occipital region—cranium in general; the face into the parotideal region—nasal region—orbitary region—zygomato-maxillary region—masseteric region—genial region—mental region—labial region, comprising the superior and inferior lips—olfactory region—buccal cavity or region—parts constituting the tongue—pharyngeal region or cavity. Each of the regions again is treated of in reference to its "constituent parts," arranged in the following order. This is the frontal region.

1. The Skin.
2. The Cellulo-adipose Layer.
3. The Muscles and Aponeurosis.
4. The Pericranium.
5. The Arteries.
6. The Veins.
7. The Lymphatic Vessels.
8. The Nerves.
9. The Skeleton.

From this it will be seen that the plan is comprehensive, the details minute. The author promises scrupulous accuracy, having first consulted nature and drawn up his own observations, then compared them with those of authors of eminence, accepted them forthwith if they agreed, and if they did not, having again referred to nature to settle the dispute. This is the best way of arriving at accuracy that we know of.

With regard to the execution we can only say that from the little that we have seen of the volumes, we should pronounce a very favourable opinion. We shall select a specimen, not that it is the best, but that others, of more value, are too long for extraction.

"*The Isthmus of the Throat.*

This opening is formed, inferiorly, by the dorsal surface of the tongue; superiorly, by the velum and velum pendulum palati; and laterally, by the pillars of the velum.

The velum palati is a prolongation of all the soft tissues of the superior paries of the mouth and inferior of the nasal fossæ, and also includes a certain number of muscles which determine its movements. We consequently find in it, in the first place, a thick, slightly extensible, and lacerable mucous membrane, which is generally of a deeper colour than that of the interior of the nose and mouth, and lined by a layer of filamentous and dense cellular tissue, in which there are a great number of very large follicles; next, another lamellated membrane, which unites the latter to the muscles. It is in the first of these tissues that purulent and œdematous infiltrations, etc. are developed, and these are the follicles which appear to be the principal seat of disease in the various affections of the velum palati. In these laminæ the principal nerves and vessels ramify; but they are unimportant in a surgical point of view. It is proper to observe, however, that it is very liberally supplied with venous capillaries; which perhaps will enable us to explain the prompt and beneficial results which some surgeons derive from touching the mucous membrane with the nitrate of silver in certain inflammations.

A man was suddenly attacked with a very acute pain in the upper part of the mouth, and, on inspection, we perceived a reddish blue spot, of the size of a two franc piece, upon the anterior surface of the velum palati, the rest of the throat being in its natural state. We touched this spot with the nitrate of silver: one hour afterwards the pain had ceased, and on the day following the redness had disappeared.

Next, the muscles: these are, the levatores palati, which draw the velum towards the nasal fossæ; the tensores palati, which widen it by drawing it horizontally, in consequence of their being reflected over the hook of the pterygoid process, forming a pulley; the palato-pharyngæus and constrictor isthmi faucium, which depress it towards the base of the tongue; lastly, the levator uvulæ, which appertains especially to the uvula. As all these muscles, with the exception of the azygos uvulæ, lie upon the sides, we may easily explain how the separation which exists between the two halves of the velum palati, when this organ is divided, is produced; but it is not so easy to account for their spontaneous approximation, in some convulsive actions of the pharynx, as is frequently observed, for example, when the *staphyloraphy* is performed. We had an opportunity of observing this phenomenon very distinctly, in a female operated upon in June by M. Roux. We in fact saw, and M. Roux directed our attention to it several times, the two lips of the division pass towards each other and come into contact, whilst this skilful surgeon was attempting to lay hold of them with the forceps or tenaculum. This fact is not satisfactorily explained to us by the known laws of muscular contraction.

The velum, like the vault of the palate, seems to be formed by the approximation of the two lateral parts. Now, if this approximation is not effected, a congenital division will be the result, which may exist alone, and thus form a species of hare-lip in the back part of the mouth; or it may be accompanied with a more or less extensive separation of the palatine suture, which being prolonged forwards, may coincide with a single or double hare-lip. It is for the purpose of removing this infirmity, heretofore considered as incurable, that M. Roux invented the *staphyloraphy*. This operation, simple and easy in itself, but delicate and tedious on account of the depth of the organs upon which it is necessary to act, has already been performed by the celebrated surgeon of *la Charité* about twenty times; and if not always with complete success, at least with a sensible amelioration in such cases as would not admit of a perfect re-union. At first view, we might apprehend that the sutures would cut through the soft parts which they must embrace; but observation has shown that this does not happen; and this may be accounted for by the compact texture of the mucous membrane, and especially of its cellular tissue, which is almost fibrous; by that of the circumflexus palati, which becomes aponeurotic in this situation; and lastly, by that of the azygos uvulæ, the entire body of which is comprised within the loop of the ligature. When the attachment of the velum palati to the posterior margin of the vault participates in the malformation, it may hinder the re-union of the soft parts: in such a case, M. Roux makes a transverse incision, and separates this membrane on each side, by carrying it along the posterior border of the floor of the nostrils; after which there is no obstacle to the approximation of the scarified edges of the wound. This operation is, without contradiction, one of the most brilliant conquests of the surgery of the nineteeenth century.

The free margin of the velum palati is prolonged in its middle by a conical eminence, the length of which varies considerably. This small body which is indirectly attached to the posterior spine of the nasal fossæ, is called uvula, and does not exist in animals, unless it is the ape, in which it is very small. It contains the same elements as the velum palati, and its figure is moulded upon the azygos uvulæ muscle, which retracts and partly elevates it. The mucous membrane forms the greater portion of it; indeed, with the follicles, it constitutes the whole of the inferior half of its free portion. These follicles are so large and numerous that they form a thick layer which gives to the uvula a very distinct glandular appearance. M. Lisfranc says that there are three in particular at the extremity of this organ, which are very large. It is to their swelling, to the inflammation of the cellular tissue which envelops them, or to serous effusions into the laminæ of the mucous membrane, that what is vulgarly called the falling down of the palate is to be attributed. Of whatsoever nature this elongation may be, it occasions much inconvenience, and a troublesome cough, in consequence of the irritation which it keeps up in the throat by titillating the tongue. In all these cases, if it is not indurated, or of very long standing, cauterizing it with the nitrate of silver will seldom fail to cure. But should this means prove unsuccessful, we must resort to excision as the only resource which holds out a chance of success. For this purpose the scissors invented by Percy, having one of its blades a little longer than the other, and bent towards the end transversely, so as to prevent the organ slipping from between the blades, are very serviceable; or we may seize the end of the uvula with a hook or forceps, and then snip it off with a blunt pair of scissors. When the tumefaction of the uvula is very great, the greater part of it may be removed without inconvenience. Surgeons have frequently supposed that they have cut it off at its root, when only that portion of it which is below the azygos was actually removed, and the congestion of the tissues being relieved by the operation, they resume their regular position; and the uvula then appearing almost of its natural length, some have supposed that it was regenerated.

Upon each side of the uvula, the border of the velum palati forms an arch which bifurcates in descending, in order to form the pillars, and thus constitute the lateral parts of the pharyngeal isthmus. The anterior branch or pillar includes the glosso-pharyngæus muscle and is lost upon the side of the tongue; the posterior descends into the lateral paries of the pharynx, and seems to go to attach itself to the body of the os hyoides: this encloses the palato-pharyngæus muscle. These two pillars consequently leave between them a triangular space, the base of which is below, and in which the tonsils are situated. These last organs are composed of a great number of mucous follicles, intimately adherent to the mucous membrane, which also sends numerous processes between them, uniting them to each other. The amygdalæ are subject to two species of inflammation; that is to say, the inflammatory state may be developed in the surface of the mucous tissue, which generally produces factitious membranes of different species, which may be mistaken for ulcers and even gangrene; or the inter-follicular and sub-mucous cellular tissue may be the principal seat of the inflammation. In the latter case, phlegmonous abscesses are disposed to form, and when they frequently recur they may occasion induration. As the use of the knife is frequently required in inflammations of the tonsils and their consequences, it is necessary to know the exact relations of this gland, especially its external part. It is, in fact, in this situation that it is approximated to the internal carotid artery, from which it is separated, in the natural state, only by the constrictor of the pharynx, some cellular tissue, nervous filaments and a complicated venous plexus. In general, the artery is eight or ten lines behind and external to the gland; so that in plunging the bistoury between the pillars of the velum palati, it would be easier to strike this vessel when the tonsil is in a state of tumefaction, as it is then carried near to the artery. In order to avoid this terrible accident, which must almost inevitably be mortal, it would be better to direct the point of the instrument more towards the pharynx, than towards the ramus of the jaw. Notwithstanding Burns relates one example, M. Portal a second, and Béclard a third, this accident must be very rare, and can only happen to those who, by distraction or some other cause, have entirely forgotten the anatomy of the posterior fauces. In the extirpation of this gland when scirrhous, there is still less risk, because the organ being drawn forwards by the forceps, it is scarcely possible, in cutting it out with the scissors or bistoury, to dip so deep as the artery. But it must not be forgotten that, during this operation, the velum palati, the pillars and tongue are alternately elevated and depressed, so that the gland appears prominent at one moment and at the next depressed: hence the advantage of using the scissors, for although the probe-pointed bistoury may be conducted by a dextrous hand, it will be liable to wound other organs unnecessarily. The hæmorrhage which follows this operation is sometimes considerable, and proceeds from the very complex net-work which is formed in the tonsil by the superior and inferior palatine arteries, and to the tonsillary circle which results from their anastomoses being of considerable volume. But more frequently the blood is discharged from numerous large veins, which form a species of plexus externally and against the posterior wall of the pharynx.

It is in the same depression in which the tonsils are lodged, and upon the tonsils themselves, that syphilitic ulcers of the throat are generally developed; it is also upon these organs that croupal concretions begin to form in the greater proportion of such cases; therefore these parts should be closely inspected when we have the least suspicion of these diseases." 128.

We need hardly say that we recommend the work to the attention of the profession.

